# When to operate after SARS-CoV-2 infection? A review on the recent consensus recommendation of the DGC/BDC and the DGAI/BDA

**DOI:** 10.1007/s00423-022-02495-8

**Published:** 2022-03-21

**Authors:** J. Noll, M. Reichert, M. Dietrich, J. G. Riedel, M. Hecker, W. Padberg, M. A. Weigand, A. Hecker

**Affiliations:** 1 Department of General, Visceral, Thoracic, Transplantation and Pediatric Surgery, University Hospital of Giessen, Rudolf-Buchheim-Strasse 7, 35392 Giessen, Germany; 2 Department of Anesthesiology, University Hospital of Heidelberg, Heidelberg, Germany; 3 Medical Clinic II, University Hospital of Giessen, Giessen, Germany

**Keywords:** Postponement, Operation, Surgery, COVID-19, SARS-CoV-2, Pandemic

## Abstract

Since the eruption of the worldwide SARS-CoV-2 pandemic in late 2019/early 2020, multiple elective surgical interventions were postponed. Through pandemic measures, elective operation capacities were reduced in favour of intensive care treatment for critically ill SARS-CoV-2 patients. Although intermittent low-incidence infection rates allowed an increase in elective surgery, surgeons have to include long-term pulmonary and extrapulmonary complications of SARS-CoV-2 infections (especially “Long Covid”) in their perioperative management considerations and risk assessment procedures. This review summarizes recent consensus statements and recommendations regarding the timepoint for surgical intervention after SARS-CoV-2 infection released by respective German societies and professional representatives including DGC/BDC (Germany Society of Surgery/Professional Association of German Surgeons e.V.) and DGAI/BDA (Germany Society of Anesthesiology and Intensive Care Medicine/Professional Association of German Anesthesiologists e.V.) within the scope of the recent literature. The current literature reveals that patients with pre- and perioperative SARS-CoV-2 infection have a dramatically deteriorated postoperative outcome. Thereby, perioperative mortality is mainly caused by pulmonary and thromboembolic complications. Notably, perioperative mortality decreases to normal values over time depending on the duration of SARS-CoV-2 infection.

## Pathophysiology of COVID-19 (coronavirus disease 2019)

During SARS-CoV-2 (severe acute respiratory syndrome coronavirus type 2) pandemic a large amount of elective surgical operations had to be postponed or even cancelled [1, 2]. Literature reveals that several subgroups of patients (e.g. emergency and oncologic surgery) were inadequately treated caused by increased time-to-diagnosis and time-to-intervention [3, 4]. Furthermore, an enormous economic disaster for surgical departments was caused by a lack of intensive care and surgical capacities [1]. Surgeons have to learn about and to work with the SARS-CoV-2 infection and with patients with COVID-19 as an accompanying part of patients’ history and perioperative management. Only a few percent of patients could become infected with SARS-CoV-2 in the perioperative setting [5, 6]; however, the rate of unreported cases could be higher due to failure in patient testing and clinically silent infections [7, 8]. It is a known fact that SARS-CoV-2 and its variants will be part of everyday work in the next decade [9]. Since the first cases in December 2019 the SARS-CoV-2 pandemic [10], the virus spread quickly [11] and became a global health crisis [12]. On March 11th, 2020, the World Health Organization (WHO) declared the SARS-CoV-2 eruption to a worldwide pandemic [13]. As typical for coronaviruses, SARS-CoV-2 infection initiates by binding of the spike protein (S protein) to the cellular ACE2 (angiotensin-converting enzyme 2) receptor [14–16] after priming by the transmembrane serine protease (TMPRSS2) [14]. The ACE2 receptor is expressed ubiquitously, but mainly in the lung, kidney, gastrointestinal (GI) tract [17] and the heart [18]. Extrapulmonary manifestations [19] of SARS-CoV-2 infection could be explained by the enzyme Furin, which promotes the SARS-CoV-2 attachment and which is expressed in a variety of organs [20]. Depending on the immune status of the infected host, the manifestation and the course of the disease can be heterogenous in the patient population: The spectrum reaches from asymptomatic patients, mild cough and fever up to critical illness with intensive care treatment and total disruption of the lung parenchyma and other organ manifestations and consecutive organ failure [21–23]. Different mortality rates are reported in the literature [21–24], but sometimes the case-fatality rate is also reported [25]. The data vary, for example in different countries, in the timing of the pandemic (different waves), depending on the age of the patient [26] and between virus variants [27]. According to the Robert-Koch Institute (RKI), a total of 1.8% of all persons for whom confirmed SARS-CoV-2 infections have been transmitted in Germany have died in association with COVID-19 disease (23 November 2021) [23]. Severe courses of SARS-CoV-2 infection are particularly dangerous, with a case-fatality rate of approximately 49% [25]. In the first wave of COVID-19 in Germany, the course of the disease was mainly mild (80% of cases). The mortality was reported to be 5.6% of all laboratory-confirmed cases, varying between 0 and 30% depending on age [28]. In the second wave, a higher mortality rate was detected worldwide due to the emergence of different virus variants [29]. Severely infected COVID-19 patients suffer from severe respiratory failure in most cases and die frequently due to acute respiratory distress syndrome (ARDS) [30]; thus, ventilated patients have a particularly high mortality risk [31]. One possible explanation for the increased incidence of severe pulmonary complications such as ARDS is the degradation of ACE2 during SARS-CoV-2 infection [32], because of the loss of the lung-protective effect attributed to ACE2 [33]. Severe courses are furthermore accompanied by extrapulmonary manifestations like thromboembolic events [34–37]. In addition to an increased incidence of acute ischemic strokes [38], the thrombus burden of ST-elevation myocardial infarction (STEMI) is also increased in cases with concomitant SARS-CoV-2 infection [39]. Intensive research on SARS-CoV-2 revealed that the virus frequently leads to neurologic disorders like encephalopathy, stroke or cerebral seizure [40], musculoskeletal weakness and impaired concentration, especially in elderly patients [41].

Not only short-term but also long-term damage have been reported, especially in patients following severe COVID-19 infection with critical illness [42], but also in younger patients with a mild course of infection [43]. There is currently an undifferentiated terminology to describe persistence or reemergence of symptoms after SARS-CoV-2 infection, summarized as “Long Covid” or “post-COVID-19 syndrome” [44]. Therefore, National Institute for Health and Care Excellence (NICE) guidelines define “acute COVID-19” as COVID-19-associated symptoms lasting up to 4 weeks after infection and “ongoing symptomatic COVID-19” as COVID-19-associated symptoms lasting 4–12 weeks after infection. “Post-COVID-19 syndrome” is characterized by COVID-19-associated symptoms lasting longer than 12 weeks after infection. The term “Long Covid” is used for both “ongoing symptomatic COVID-19” and “post-COVID syndrome” [45]. These terms are used in the same way in the German guideline on Long Covid [46]. Risk factors for the development of Long Covid include older age and higher body mass index and female sex. The symptoms of fatigue, headache, dyspnea, hoarse voice and myalgia that occur during the first week of infection crystallized as good predictive factors for the development of Long Covid [47]. So far, the literature does not provide a reliable estimation for the incidence of Long Covid [23, 46]. In Long Covid, the most common manifestation is chronic fatigue syndrome [42, 48–50]. The causes for the development of a Long Covid are still unclear. However, pathologic and persistent systemic inflammation in response to viral and antigenic remnants, as well as the ongoing persistence of SARS-CoV-2 infection, are discussed in the pathophysiology of the disease [51, 52]. Other possible causes such as immune cell dysfunction with the development of autoimmune processes and alteration of the microbiome of the gastrointestinal tract are also still a matter of debate [53, 54]. In the following, we will present the evidence for possible existing additive negative effects of passed SARS-CoV-2 infection on the outcome of surgical patients. The optimal timing for elective surgery will be highlighted. In addition, the applications of risk scores and useful primary prophylactic treatments to prevent postoperative complications are discussed thoroughly.

## Possible synergies between SARS-CoV-2 infection and surgical intervention on the immune system

**Fig. 1:**
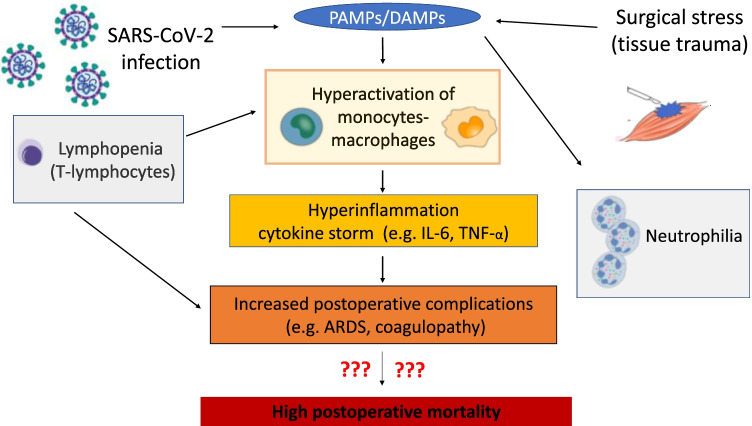
Common immunomodulatory effects of SARS-CoV-2 infection and surgical therapy on postoperative mortality. Both SARS-CoV-2 infection and surgical therapy lead to hyperactivation of macrophages through tissue damage of various causes, which first leads to local hyperinflammation. In the following course, a systemic cytokine storm may occur. In this line, lymphopenia and neutrophilia are induced. These SARS-CoV-2 driven effects on the immune system negatively influence on postoperative immune competence of patients and lead to severe postoperative complications such as ARDS, sepsis and thromboembolism. The question now concerns the impact of perioperative SARS-CoV-2 infection on postoperative mortality. ARDS, acute respiratory distress syndrome; PAMPS, pathogen-associated molecular patterns; DAMPS, damage-associated molecular patterns; IL-6, interleukin-6, TNF-α, tumour necrosis factor-α (modified from [75]; Icons from [76, 77])

The most common postoperative complications and major causes of death after surgery are postoperative infections [55, 56] and thromboembolic events [57–59]. Surgery results in a hyperinflammation-induced procoagulant status in the perioperative phase due to impairments of the immune system [60]. Notably, not only in postoperative patients, but also in patients with severe SARS-CoV-2 infection, a frequent cause of death is caused by modulation of the immune system, leading to severe pulmonary (ARDS) [10, 61, 62] and thromboembolic complications [34, 36, 63–69]. Thromboembolic complications are not only characterized by the formation of microthrombi in the lungs [70]. Rather, COVID-19 is a systemic vascular disease with multiple manifestation sites [71, 72] and platelet activation [73]. In severe SARS-CoV-2 infection, this procoagulant state is diagnostically associated with elevated D-dimer levels [64, 74]. The common immunomodulatory effects of SARS-CoV-2 infection and surgical therapy are depicted in Fig. 1.

Initially, SARS-CoV-2 is recognized by endothelial cells and monocytes/macrophages through its viral RNA, known as PAMP (pathogen-associated molecular patterns). PAMPs activate monocytes/macrophages and cause dysfunctional proinflammatory cytokine response leading to a cytokine storm with consecutive hyperinflammation [76, 78–82]. The level of cytokine release in these cases correlates positively with the severity of the disease [79, 83–85]. Tissue damage induced by hyperinflammation in the context of SARS-CoV-2 infection and iatrogenic tissue damage in the setting of surgery each result in the release of DAMPs from the damaged cells [86, 87]. Iatrogenic tissue damage may also cause hypersecretion of proinflammatory cytokines resulting in a vicious circle [85, 88]. In the initial acute stage of SARS-CoV-2 infection, there is also a characteristic lymphopenia [61, 78, 79, 89] and the extent of initial lymphopenia by itself often correlates with the severity of the SARS-CoV-2 infection [90]. For this reason, severe courses have been observed especially in elderly patients [89] with an already weakened immune system [91]. Hyperinflammation may also be aggravated by the presence of lymphopenia, as the reduced number of T lymphocytes may not adequately inhibit macrophages in their proinflammatory activity. Lymphopenia also exists postoperatively as part of the systemic stress response, but it differs from SARS-CoV-2-induced lymphopenia because of other pathomechanisms (e.g. endocrine responses involving cortisol release [92]). Another common immunomodulatory reaction that can be triggered by both SARS-CoV-2 infection and surgical therapy is neutrophilia [22, 93]. In the synopsis of the immunogenic changes just presented, an increased neutrophil-to-lymphocyte ratio (NLR) can be detected especially in severe SARS-CoV-2 infections of critically ill patients [89] and can also be used as a predictive marker for postoperative complications [94].

In summary, synergistic immunopathologic mechanisms of SARS-CoV-2 and surgery are to be expected in patients with perioperative SARS-CoV-2 infection.

**Fig. 2:**
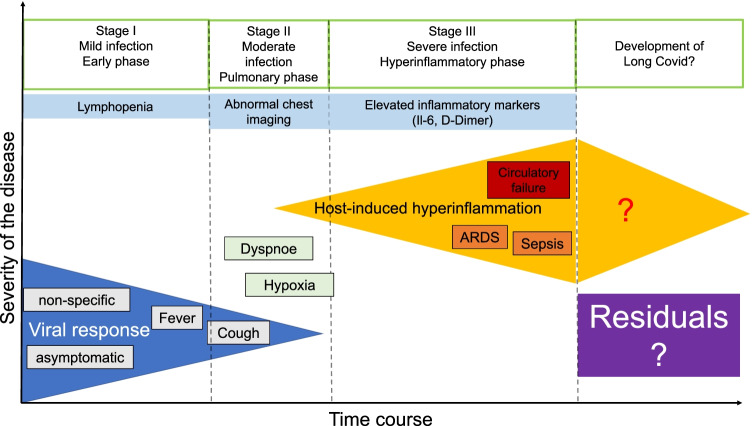
The different diseases phases of SARS-CoV-2 infection in relation to the severity of COVID-19. The initial phase is characterized by mild infection with cough and fever or even presents asymptomatically. Blood examinations might give evidence for lymphopenia and neutrophilia. The prognosis at this stage is very good. In case of progression of the infection, a transition to a pulmonary phase with clinical and morphological development of pneumonia can be found, which makes frequently hospitalization necessary. The prognosis depends on the severity of pulmonary function impairment or respiratory insufficiency and comorbidities of the affected patients. Transition to the 3rd phase results in the development of a systemic extrapulmonary syndrome with a systemic increase in proinflammatory markers. The prognosis is poor due to the development of sepsis with multiple organ failure and/or ARDS (modified from [96])

For surgeons planning elective surgical interventions, it is important to know and to learn about the time course and the possibility of prolonged dysfunction of the immune system in Long Covid: In the early phase of SARS-CoV-2 infection, there is a mild inflammation characterized by a high viral load in the frequently asymptomatic or by coughing and fever, mildly symptomatic patient. Subsequently, moderate infection with most common pulmonary manifestation and dyspnea may occur. Severe courses are characterized by an endogenous hyperinflammatory response, that frequently lead to ARDS, sepsis and even circulatory failure. A prolonged course of SARS-CoV-2 infection with a very slow regression of the immunologic activation [95] and the development of prolonged COVID-19 Long Covid should be considered for the affected patients when planning elective surgical procedures (s. Fig. 2) [96].

The different stages and varying clinical courses of SARS-CoV-2 infection underline the complexity of planning surgical interventions with additional (surgical) trauma in the affected patients. An increasing amount of literature has been published in that field, which has to be considered for the safety of future patients during the pandemic and which is reviewed in the following.

## Increased postoperative mortality in cases of perioperative SARS-CoV-2 infection

At the beginning of the SARS-CoV-2 pandemic, most guidelines focused on perioperative management and hygiene precautions of SARS-CoV2-positive patients. The intention was to control the spread of infection and protect other patients and healthcare workers from infection [97–99]. However at this time, due to missing studies, no recommendations could be made for the optimal time period between SARS-CoV-2 infection and elective surgery. Up to date, there are many publications, analyzing the perioperative mortality of patients with perioperative SARS-CoV-2 infection [100] and giving an evidence-based statement on postponing elective surgical interventions [101]. Table 1 summarizes some selected publications from the onset of the pandemic to the appearance of studies that examine the time interval in more detail [8, 102–110].

**Table 1:** Summary of selective publications on perioperative outcome after SARS-CoV-2 infection

Authors	Title	Journal	Publication date	Country	Study design	Period of surgery	Sample size	Diagnosis/start of COVID-19 infection	Examined perioperative period (detection of SARS-CoV-2 infection)	Mortality	Most common complications	Recommendation distance from operation to SARS-CoV-2 infection	Some limitations
Lei et al. [8]	Clinical characteristics and outcomes of patients undergoing surgeries during the incubation period of COVID-19 infection	EClinicalMedicine (The Lancet)	April/2020	China (Hubei province, Wuhan)	Retrospective cohort study	01/2020–02/2020	34	Onset of clinical symptoms	During the incubation period of COVID-19 infection	20.5% (patients with perioperative COVID-19 infection), no comparison group	Pneumonia, ARDS, secondary infection	Preoperative quarantine period, exclusion of new COVID-19 infection	Small sample size, PCR tests preoperative not performed as standard
CovidSurg Collaborative [102]	Mortality and pulmonary complications in patients undergoing surgery with perioperative SARS-CoV-2 infection: an international cohort study	The Lancet	May/2020	international (24 countries, predominantly Europe and North America)	Retrospective cohort study	01/2020–03/2020	1128	PCR test or clinical suspicion or radiological signs	7 days preoperative to 30 days postoperative	30-day mortality rate: 23.8% (perioperative COVID-19 infection), 43.1% (emergency surgery, postoperative COVID-19 diagnosis, pulmonary complications), no comparison group	Pulmonary complications	Generous postponement of operations, balancing the consequences of postponed surgery and expected postoperative mortality with perioperative COVD-19 infection (risk factors: male and advanced age)	Not always PCR test used for diagnosis
Kahlberg et al. [103]	Vascular surgery during COVID-19 emergency in Hub Hospitals of Lombardy: experience on 305 patients	European Journal of Vascular & Endovascular Surgery	November/2020	Italy (Lombardy)	Prospective study	03/2020–04/2020	305	PCR test and clinical suspicion with radiological signs	Pre- and postoperative	COVID vs non-COVID patient: In-hospital mortality: 25% vs 6%, Elective: 20.0% vs 2.8%, Emergent: 27.9% vs 13.2%	Multiorgan failure, respiratory failure	In surgical planning: consider COVID-19 infection as a negative prognostic factor (pulmonary and vascular complications)	Not always PCR test used for diagnosis
Mi et al. [104]	Characteristics and Early Prognosis of COVID-19 Infection in Fracture Patients	The Journal of Bone And Joint Surgery	May/2020	China (Hubei province, Wuhan)	Retrospective cohort study	01/2020–02/2020	10	PCR test and/or radiological signs	COVID-19 infection before admission, postoperative	Of 2 patients with COVID-19 infection detected by PCR test and surgical treatment 1 died	Pulmonary complications	Surgical treatment should be carried out cautiously or non-operative care should be chosen	Very small sample size, not always PCR test used for diagnosis
COVIDSurg Collaborative [105]	Delaying surgery for patients with previous SARS-CoV-2 infection	British Journal of Surgery	November/2020	International (16 countries, predominantly Italy, UK, Spain)	Prospective cohort study	01/2020–03/2020	122	PCR test	preoperative	30-day mortality 3.4% (all patients with positive PCR test), 7.7% (1–2 weeks after positive PCR test), 3–4% (2–4 weeks after positive PCR test), 0% (> 4 weeks after positive PCR test), no comparison group	Pulmonary complications (10.7% COVID-19 infection vs 3.6% no COVID-19 infection)	Postponement of surgery > 4 weeks after positive PCR result	Small sample size
Doglietto et al. [106]	Factors associated with surgical mortality and complications among patients with and without coronavirus disease 2019 (COVID-19) in Italy	JAMA Surgery	June/2020	Italy (Brescia)	Retrospective cohort study	02/2020–04/2020	123	PCR test and/or radiological signs (chest radiography and/or computed tomography)	Preoperative or within 1 week after surgery	COVID vs non-COVID patient: 30-day mortality: 19.51% vs 2.44%	Pulmonary and thrombotic complications	Postpone surgery if possible, because of increased mortality has been demonstrated	Not always PCR test used for diagnosis, single-center study
Catton et al. [107]	Planned surgery in the COVID-19 pandemic: a prospective cohort study from Nottingham	Langenbeck’ s Archives of Surgery	May/2021	UK (Nottingham)	Prospective cohort study	03/2020–04/2020	597	PCR test confirmed suspected cases (temperature measurement and questionnaire or imaging)	2 days preoperative to 30 days postoperative	30-day mortality: 0.7% (all postoperative patients)vs 25% (postoperative patients with COVID-19 infection)	No information	Patient should be informed about increased mortality rate in COVID-19 infection after surgery. Urgent and cancer operations can take place with a low incidence of COVID-19 infection	Not always PCR test used for diagnosis, mortality not clearly attributable to COVID19 infection (e.g. palliative situation) small number of COVID-19 diagnosis or suspected COVID-19 infections (18 patients)
Jonker et al. [108]	Perioperative SARS-CoV-2 infections increase mortality, pulmonary complications and thromboembolic events: a Dutch, multicenter, matched-cohort clinical study	Surgery	September/2020	Netherlands	Retro- and prospective cohort study	02/2020–06/2020	558 screened for the study, 503 included in data analysis	PCR test or clinical suspicion plus radiological signs (computed tomography of the chest)	30 days before surgery or within 30 days postoperatively	COVID vs non-COVID patient: 30-day mortality: 12% vs 4%	Pulmonary and thromboembolic complications	Postponing elective surgeries and, if possible, emergency surgeries, altered protocols of thromboembolic prophylaxis	Not always PCR test used for diagnosis
COVIDSurg Collaborative & GlobalSurg Collaborative [109]	Timing of surgery following SARS-CoV-2 infection: an international prospective cohort study	Anaesthesia	March/2021	International (116 countries)	prospective cohort study	10/2020	140 231	PCR test or rapid antigen test or computed tomography of the chest or antibody test or clinical suspicion	Preoperative	30-day mortality (weeks after COVID-19 diagnosis): 9.1% (0–2 weeks), 6.9% (3–4 weeks), 5.5% (5–6 weeks), 2% (> 7 weeks), 1.4% (no preoperative COVID-19 infection)	Pulmonary complications	Postpone surgery > 7 weeks after COVID-19 infection, longer for patients with persistent symptoms	Not always PCR test used for diagnosis
National emergency laparotomy audit [110]	The impact of COVID-19 on emergency laparotomy – an interim report of the national emergency laparotomy audit	Royal College of Anaesthetists	March/2021	England and Wales	Retrospective cohort study	03/2020–09/2020	10,546	PCR test or clinical suspicion	Pre- and postoperative	COVID vs non-COVID patient: 30-day mortality: 12.5% vs 7.2%	No data	Due to increased postoperative mortality with COVID-19 infection, high-risk patients should be offered alternative/conservative therapies	Not always PCR test used for diagnosis

*PCR*, polymerase chain reaction [8, 102–110]

The studies were published from 2020 to mid-2021 and examine only a small time interval in 2020, which only represents the early global onset of the pandemic [8, 102–110]. Unfortunately, there are some limitations of the 10 studies, shown in Table 1. The majority of studies (7 out of 10 studies) is not international, but focus only on certain countries [8, 103, 104, 106–108, 110]. Only half of the studies were prospectively conducted [103, 105, 107–109]. The studies show a high degree of heterogeneity in terms of:

- The contingent of surgeries investigated (investigation of heterogeneous surgeries [8, 102, 106, 108, 109] versus investigation of a specific type of surgery [103–105, 107, 110],
- The high range in case-loads of the studies (ranging from 10 to 140,231 patients),
- The modality of SARS-CoV-2 diagnosis (e.g. clinical symptoms [8], clinical suspicion and/or chest imaging with or without confirmation by either rapid antigen or PCR-testing
- The perioperative period was studied (many studies without precise data [8, 103–105, 109, 110], with the largest period 30 days before to 30 days after surgery [108].

Some studies report only the mortality of patients with pre- or perioperative SARS-CoV-2 infection without a comparison group (mortality of patients with postoperative SARS-CoV-2 infection and ICU stay: 20.5% [8], overall 30-day mortality in patients with perioperative SARS-CoV-2 infection: 23.8% [102], overall 30-day mortality in patients with preoperative SARS-CoV-2 infection and different surgical time points after confirmed SARS-CoV-2 infection: 3.4% [105]. Mortality of patients with perioperative SARS-CoV-2 infection compared with patients without SARS-CoV-2 infection has been investigated in 4 studies and was significantly increased in cases with perioperative SARS-CoV-2 infection [103, 106, 108, 110]. Only two studies investigated the impact of the selected time interval from diagnosis of SARS-CoV-2 infection to surgery on postoperative mortality. The majority of the presented studies observed an increase in postoperative pulmonary and thromboembolic complications [8, 102–106, 108, 109] and came to the conclusion that elective surgical interventions should be postponed [102, 104–106, 108, 109]. Herein, a longer time interval between SARS-CoV-2 infection and surgery reduces postoperative mortality [105, 109]!

In the landmark trial from the COVIDSurg Collaborative and the GlobalSurg Collaborative dealing with “Timing of surgery following SARS-CoV-2 infection” the 30-day mortality rates in more than 140,000 patients (non-COVID-19 (97.8%) versus former COVID-19 (2.2%) patients) were depicted. While 30-day mortality was 1.5% (95% CI 1.4–1.5) in patients without SARS-CoV-2 infection, mortality was increased in SARS-CoV-2-positive patients depending on the time to surgery after infection. When surgery was performed 0–2 weeks after SARS-CoV-2 infection, mortality was increased (odds ratio (95% CI) 4.1 (3.3–4.8)) and decreased 5–6 weeks after SARS-CoV-2 infection (odds ratio (95% CI) 3.6 (2.0–5.2)). Finally, patients operated on 7 weeks or longer after infection had mortality similar to that of uninfected patients. These results are also evident in the different subgroups classified by age, ASA physical status, grade (on the basis of the Bupa schedule of procedures in minor and major) and urgency of surgery elective vs emergency. Indications for surgery were divided into “trauma”, “benign”, “cancer” and “obstetrics”.

Interestingly, results revealed that symptomatic patients with a prolonged COVID-19 disease without resolution of symptoms had persistently increased mortality rates and might benefit from a further postposition of the elective surgical intervention. This important trial was one of the largest global surgical studies ever conducted. The GlobalSurg Collaborative and COVIDSurg Collaborative give mandatory information on postoperative mortality after SARS-CoV-2 infection and furthermore demonstrated a time-dependent decrease in unfavourable patient outcomes after SARS-CoV-2 infection, which might be a recommendation and transferred to future patients during the global pandemic.

### Planning of surgery is an individual and interdisciplinary decision

Before the SARS-CoV-2 pandemic, elective operations were postponed in children with recent severe upper respiratory tract infection [111]. The rationale was that postoperative respiratory complications were otherwise observed more frequently [112, 113]. In adults, a former respiratory infection within 1 month prior to surgery was also identified as a risk factor for postoperative pulmonary complications and increased mortality [114]. In contrast to “classical” respiratory diseases, the reasons for postponing elective surgery in the current COVID-19 situation are more complex. Operations are postponed not only because of increased postoperative complication rates, but also because of the risk and the fear of nosocomial SARS-CoV-2 infection, in-hospital spreading [5] and reduced intensive care capacities due to the pandemic as well as reserved intensive care capacities for critical ill COVID-19 patients during the phases with high incidences [115]. When deciding whether elective operations can be postponed, the indication and urgency of the operation must be considered [116, 117]. Evaluating the urgency of the operation becomes particularly difficult in patients with malignant diseases. Cancer progression and systemic spreading may lead to increased cancer-related mortality, if oncologic surgery (and medical oncologic treatment) is delayed [4]. On the other hand, cancer patients with perioperative SARS-CoV-2 infection in particular often require postoperative monitoring and ventilation at an ICU and show higher postoperative mortality [118]. Not only the type of surgery (e.g. high mortality rate of almost 40% after thoracic surgery [119]) but also the severity of COVID-19 infections (with potentially long duration until complete rehabilitation and functional recovery) and the development of a Long Covid should be taken into account for the treating surgeon. Existing comorbidities of COVID-19 patients associated with a poorer prognosis are, e.g. male gender, diabetes mellitus type II, advanced age and cardiovascular disease [30, 120–128]. Obese patients in particular are at increased risk of SARS-CoV-2 infection with a severe course [129]. This is particularly evident in ICU patients with COVID-19 [130].

Many efforts were made to analyze the impact of the time interval to a passed SARS-CoV-2 infection on postoperative mortality. On May 12th 2021, the consensus recommendation of the DGC/BDC as surgical representatives and DGAI/BDA as anesthesiologic representatives was published on the timing of planned surgical interventions after SARS-CoV-2 infection [131]. This recommendation was mainly based on the COVIDSurg Collaborative and GlobalSurg Collaborative trial mentioned above (Table 1, [109]). If possible, any planned operation should be performed seven weeks after the beginning of COVID-19 infection at the earliest convenience. This is not the case, if COVID-19 symptoms are persisting, as the COVIDSurg Collaborative and GlobalSurg Collaborative trial revealed a “normal” mortality only in patients 7 weeks after the diagnosis of COVID-19, if they are no longer symptomatic! With persistent symptoms, increased mortality was seen in patients operated 7 weeks after SARS-CoV-2 infection, thus this patient collective will benefit from a delay of at least 7 weeks. Be alerted, that the study underlying this German society recommendation refers to the suggested time interval not only to clinical symptoms, but to disease detection, where the patient does not necessarily have to be symptomatic [109]. Furthermore, it should be noted that preoperative vaccination is recommended in patients without a history of SARS-CoV-2 infection [131, 132]. An interval of at least 1 week was recommended between vaccination and elective surgery in order to distinguish possible consequences of vaccination from postoperative complications. However, to ensure that a competent immune response has occurred before surgery, the interval should be extended to 2 weeks [131, 133].

**Fig. 3:**
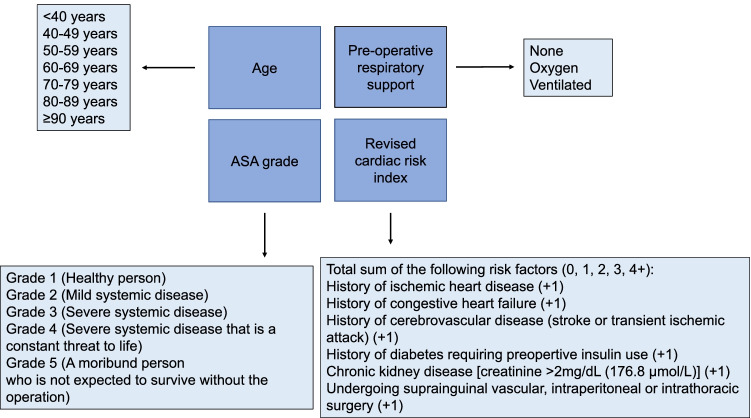
The CovidSurg Mortality Score. To estimate postoperative mortality, age, ASA and pulmonary and cardiac comorbidities are considered. Modified from https://covidsurgrisk.app and [140]

**Fig. 4:**
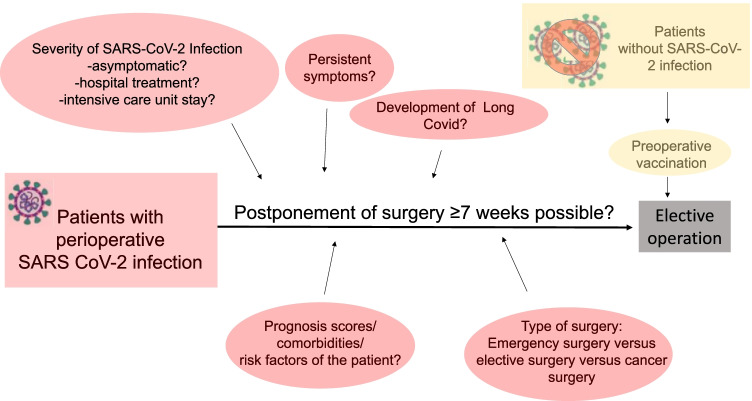
Individual and interdisciplinary factors in elective surgery planning. Summary of individual and interdisciplinary factors influencing the planning of operations in patients with and without perioperative SARS-CoV-2 infection. The SARS-CoV-2 icons by [76]

The DGCH/BDC and DGAI/BDA recommendation follows, with minor deviations, the recommendation from the Royal Australasian College of Surgeons (RACS) Victorian State Committee. On August 5th, 2020, they already recommended a symptom-free interval of 8 weeks before elective surgery [134]. Previous recommendations from the American Society of Anesthesiologists (ASA) dated December 8th, 2020, note that the time interval between symptoms and elective surgical procedures depends on the severity of the disease and should be between 4 and 12 weeks [135]. The aforementioned period of 12 weeks is supported by a study by Hsieh et al. It shows that the reconvalescence phase is 3 months after influenza-associated ARDS [136]. Prediction models already exist that estimate the mortality of COVID-19 infection [128, 137–139]. One of the first measurements for the planning of surgery and to predict mortality in patients with or after COVID-19 is *COVIDSurg Mortality Score*. It takes into account age, ASA grade, preoperative oxygen demand and cardiovascular comorbidities and is freely available under https://covidsurgrisk.app [140] (s. Fig. 3). For a comprehensive overview, Fig. 4 summarizes important aspects in the planning of elective surgery during the SARS-CoV-2 pandemic.

In conclusion, many factors should be considered when planning elective operations in patients with perioperative SARS-CoV-2 infection, including the severity and course of SARS-CoV-2 infection (e.g. asymptomatic versus severe course, outpatient versus inpatient/ICU treatment, rapid versus delayed recovery). Nevertheless, the type of surgery (e.g. emergency versus elective surgery, cancer versus benign surgery) has to be included in the decision-making [102]. Based on the recommendation of the DGCH/BDC and DGAI/BDA, the time interval is critical for postoperative mortality (postponing surgery by ≥ 7 weeks if possible) [109, 131]. Thus, the planning of surgery after SARS-CoV-2 infection is an individualized and interdisciplinary decision in times of the SARS-CoV-2 pandemic.

## Abbreviations

ACE2: Angiotensin-converting enzyme 2
ARDS: Acute respiratory distress syndrome
ASA: American Society of Anesthesiologists
COVID-19: Coronavirus disease 2019
DAMPs: Damage-associated molecular patterns
GI tract: Gastrointestinal tract
ICU: Intensive care unit
IL-6: Interleukin-6
NICE: The National Institute for Health and Care Excellence
NLR: Neutrophil-to-lymphocyte index
PAMPs: Pathogen-associated molecular patterns
RACS: Royal Australasian College of Surgeons
PCR: Polymerase chain reaction
RKI: Robert-Koch Institute
SARS-CoV-2: Severe acute respiratory syndrome coronavirus type 2
S protein: Spike protein
STEMI: ST-elevation myocardial infarction
TMPRSS2: Transmembrane serine protease
TNF-α: Tumour necrosis factor-α
WHO: World Health Organization
DGC: Germany Society of Surgery
BDC: Professional Association of German Surgeons e.V.
DGAI: Germany Society of Anesthesiology and Intensive Care Medicine
BDA: Professional Association of German Anesthesiologists e.V
